# A Non-Invasive Hemoglobin Detection Device Based on Multispectral Photoplethysmography

**DOI:** 10.3390/bios14010022

**Published:** 2023-12-30

**Authors:** Jianming Zhu, Ruiyang Sun, Huiling Liu, Tianjiao Wang, Lijuan Cai, Zhencheng Chen, Baoli Heng

**Affiliations:** 1 School of Life and Environmental Sciences, Guilin University of Electronic Technology, Guilin 541004, China; zhujianming@guet.edu.cn (J.Z.); 21082304090@mails.guet.edu.cn (R.S.); wangtj1004@163.com (T.W.); 21122202001@mails.guet.edu.cn (L.C.); 2 Guangxi Key Laboratory of Automatic Detecting Technology and Instruments, Guilin University of Electronic Technology, Guilin 541004, China; 3 Department of Urology, The First Affiliated Hospital of Jinan University, Guangzhou 510632, China; liuyxaa2021@163.com; 4 Yingde Center, Institute of Kidney Surgery, Jinan University, Guangzhou 510632, China

**Keywords:** multiwavelength photoplethysmography, hemoglobin, regression model, noninvasive detection, Pearson correlation analysis

## Abstract

The measurement of hemoglobin is a vital index for diagnosing and monitoring diseases in clinical practice. At present, solutions need to be found for the soreness, high risk of infection, and inconvenient operation associated with invasive detection methods. This paper proposes a method for non-invasively detecting hemoglobin levels based on multi-wavelength photoplethysmography (PPG) signals. AFE4490 and TMUX1109 were used to implement the low-cost collection of an eight-LED transmissive PPG signal. We used seven regular LEDs and one broadband LED (Osram SFH4737) as light sources. Additionally, a finger clip integrating multiple sensors was designed and manufactured via 3D printing to simultaneously monitor the LED–sensor distance and the pressure from the tester’s finger during PPG signal acquisition. We used a method to extract features from PPG signals using a sliding-window’s variance and an evaluation metric for PPG signals based on the AdaCost classification. Data were gathered from 56 participants from the Nephrology department, including 16 anemic patients. Pearson correlation analysis was conducted on the collected data to remove any data with a weak correlation. The advantage of using a broadband LED as a light source was also demonstrated. Several non-invasive hemoglobin regression models were created by applying AdaBoost, BPNN, and Random Forest models. The study’s results indicate that the AdaBoost model produced the best performance, with a mean absolute error (MAE) of 2.67 g/L and a correlation coefficient (R^2^) of 0.91 The study results show that the device we designed and manufactured can achieve effective non-invasive hemoglobin detection and represents a new methodological approach to obtaining measurements that can be applied in a clinical setting.

## 1. Introduction

Hemoglobin (Hb) is an essential protein in human blood that carries oxygen to the body’s organs and tissues and carbon dioxide back to the lungs for gas exchange [1]. Detecting hemoglobin levels plays a crucial role in diagnosing diseases such as anemia [2]. Anemia also requires long-term monitoring of hemoglobin levels to determine their progression. Anemia is a common disease that can cause loss of fitness, dizziness, and a lack of concentration [3]. Anemia represents an urgent public health problem. Blood is collected from a vein or capillary to test for hemoglobin, which can be inconvenient and painful and increase the risk of infection. Most anemic patients are pregnant women, children, or those recovering from major surgeries, as well as those with severe chronic diseases, who are immunocompromised, or who have coagulation disorders [3,4,5,6]. Frequent blood tests can also lead to poor compliance. Therefore, non-invasive methods of measurement need to be investigated.

The photoplethysmography (PPG) method has been widely investigated as a non-invasive hemoglobin assay because of its rapid, non-invasive method of detection and ease of use [7]. Light is absorbed by the blood, skin, muscle, bone, and pigment as it passes through tissues such as the fingers, anterior chamber, and earlobes. The regular movement of arterial blood caused by the heartbeat causes a change in light absorption, resulting in a PPG signal. This change in the PPG signal partly reflects the absorption of light by arterial blood components [8]. In the 600–1400 nm spectrum, hemoglobin and water serve as light-absorbing substances in the blood [9]. The hemoglobin concentration can be deduced by analyzing the PPG signal’s characteristics in several spectrum bands [10].

In recent years, a great deal of research has been conducted on the non-invasive detection of hemoglobin content via optical methods [11]. Yuan et al. used a 16-pixel InGaAs detector array and a planar grating spectrometer to construct a near-infrared spectrophotometric system. The authors combined the direct orthogonal signal correction (DOSC) and partial least squares (PLS) methods to obtain relative Root Mean Square Errors of Prediction (RMSEPs) of 6.16% and 6.08%, respectively [12]. However, complex spectroscopic systems can be costly. Ah-San et al. proposed a smartphone-based, non-invasive hemoglobin detection method. In this method, video images collected from human fingertips are used for non-invasive hemoglobin prediction. Although the basic hypothesis of this method works, it must be combined with more effective methods for clinical application [13]. Kesarwani et al. developed a non-invasive anemia detection system by combining current state-of-the-art computational methods with observations of palmar pallor. The proposed system ensured 93% sensitivity with Mean Squared Error (MSE) and Root Mean Squared Error (RMSE) values of 0.701g/dL and 0.698 g/dL, respectively [14]. However, its accuracy still needs to be improved. Yogesh Kumar designed a multi-wavelength pulse wave acquisition device and proposed a highly correlated multiple linear regression model with independent features; the precision of this method was 0.878 [15]. Kavsaoglu et al. used PPG signals in combination with different machine learning regression techniques. The authors concluded that using RFS feature selection methods in combination with Support Vector Regression (SVR) yielded the best results (MSE = −0.0027 g/dL) [16].

This paper explains the principle of detecting hemoglobin levels using the PPG method. To improve the traditional finger clip, we designed a finger clip integrating a PPG acquisition system, a linear displacement sensor, and a pressure sensor via 3D printing. This finger clip can simultaneously acquire eight transmitted PPG signals (the light sources include seven wavelengths of ordinary LEDs and one broadband LED), the LED–sensor distance, and the active pressure applied by the user. A window-variance-based PPG feature extraction method and a PPG signal evaluation index were also designed. In addition, several non-invasive hemoglobin regression models were created by applying Adaptive Boosting (AdaBoost), BP neural network (BPNN), and Random Forest (RF) models. The study results show that the device we designed and manufactured can achieve effective non-invasive hemoglobin detection.

## 2. Principles and Methods

### 2.1. Theory of PPG

**Figure 1:**
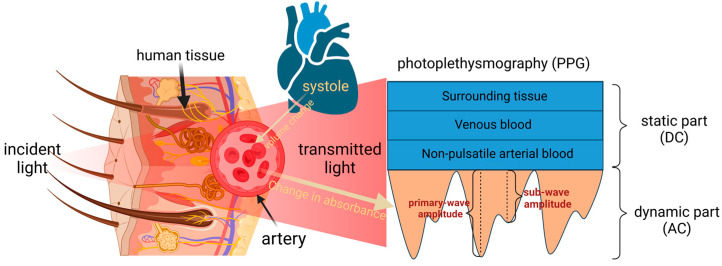
Schematic representation of PPG signal generation.

A schematic portrayal of PPG signal generation is provided in Figure 1. The process involves the modulation of light absorption by arterial blood during pulsatile flow through tissue, manifesting as a dynamic process [8]. The portion of light that passes through tissues such as fat, muscle, bone, and venous blood forms the static portion of the pulse wave. The distinctive features in the dynamic part are the primary wave and sub-wave. 

The selection of red and near-infrared light bands (625–1100 nm) as the emitted light source wavelengths was deliberate. Within this spectral range, the light demonstrates superior tissue penetration capabilities [17]. Notably, the absorption coefficients of interfering substances remain relatively modest, while the absorption peaks of deoxygenated and oxygenated hemoglobin align near 660 nm and 880 nm, respectively [18].

### 2.2. Lambert–Beer Law and Its Corollaries

The Lambert–Beer Law is the theoretical basis for the measurement of hemoglobin via PPG. This law states that when parallel monochromatic light passes perpendicularly through a homogeneous, non-scattering light-absorbing substance, the absorbance of the substance at a specific wavelength is linearly related to its concentration based on the light path [19]. The concentration and content of the light-absorbing substances in a solution can be found by measuring the solution’s ability to absorb incident light. The formula for the Lambert–Beer Law is provided in Equation (1):

(1) $$A=\mathrm{lg}(\frac{I_{s}}{I_{0}})=\varepsilon CL$$

where *A* is absorbance, $I_{s}$ is the incident light intensity, $I_{0}$ is the transmitted light intensity, *ε* is the absorbance coefficient of the measured substance, *C* is the concentration of the measured substance, and *L* is the optical range.

Human blood hemoglobin predominantly comprises oxyhemoglobin, reduced hemoglobin, and trace amounts of methemoglobin and carboxyhemoglobin. In the Near-Infrared (NIR) band, the absorption coefficients of water and plasma proteins are much smaller than those of hemoglobin [20]. Consequently, hemoglobin is the primary absorbing entity in the dynamic segment of PPG. On this basis, we obtained Equation (2). The transmitted light intensity in the static segment is expressed by Equation (3):

(2) $$I_{\mathrm{AC}}+I_{\mathrm{DC}}=I_{s}10^{(\sum_{i=1}^{n=4}\varepsilon_{Hb(i)}C_{Hb(i)}+\sum \varepsilon_{\mathrm{DC}}C_{\mathrm{DC}})L}$$

(3) $$I_{\mathrm{DC}}=I_{s}10^{\left(\sum \varepsilon_{\mathrm{DC}}C_{\mathrm{DC}})(L-\Delta L\right)}$$

where $I_{AC}$ is the light intensity in the dynamic part of the PPG; $I_{DC}$ is the light intensity in the static part of the PPG; Hb(i) denotes the hemoglobin type (including oxyhemoglobin, reduced hemoglobin, methemoglobin, and carboxyhemoglobin); and $\sum \varepsilon_{DC}C_{DC}$ is the sum of the products of the absorption coefficients for all the substances in the static part and their concentrations. Additionally, ∆L represents the change in fingertip thickness during cardiac systole and diastole.

By extracting the intensities of the dynamic and static parts, dividing $I_{AC}$ by $I_{DC}$ , and taking logarithms on both sides of the resulting equation since the dynamic part of the PPG, $I_{AC}$ , is much smaller than $I_{DC}$ , based on the Taylor series expansion, we can obtain an approximate equation, as follows (4):

(4) $$\mathrm{lg}(\frac{I_{\mathrm{AC}}+I_{\mathrm{DC}}}{I_{\mathrm{DC}}})=\mathrm{lg}(1+\frac{I_{\mathrm{AC}}}{I_{\mathrm{DC}}})\approx \frac{I_{\mathrm{AC}}}{I_{\mathrm{DC}}}=(\sum_{i=1}^{n=4}\varepsilon_{Hb(i)}C_{Hb(i)})\Delta L.$$

Individual variations such as in skin, bone, fat, and venous blood, as well as fluctuations in the emitted light source attributed to voltage and heat, are thus effectively negated. A regression algorithm enables the estimation of hemoglobin content.

In the PPG signal measurement system, in addition to the seven regular LEDs, a Broadband LED (OSRAM SFH4737) is incorporated. Transmitted light is captured by a photodiode, yielding an integral of the overlap between the photodiode’s spectral response range, the light-filtering device’s spectral range, and the LED’s emission spectrum. This value represents the summation of the transmitted light measured by numerous narrowband LEDs within a specific wavelength range. This component is introduced into the equation as a constant since the same device is employed for each measurement. For broadband LEDs, Equation (4) is reformulated as Equation (5):

(5) $$\frac{I_{AC}}{I_{DC}}=\Delta L\times \int_{Start\;point}^{End\;point}\left(\sum_{i=1}^{n=4}b_{i}\varepsilon_{Hb(i)}C_{Hb(i)}\right)$$

where the Start point–End point denotes the broadband LED emission spectral range, the filter device passband range, and the overlap between the detectable spectral range of the photodiode; these values remain constant at each wavelength, relating to the sensitivity of the photodiode and the LED emission intensity.

## 3. System Composition

We used eight LEDs and one photodiode (PD) in the process of receiving transmitted light to collect PPG signals, with the measurement site being the right index finger. 

Since the conclusions obtained from Equation (4) include the distance variation and light-traveling factor.Using a linear displacement sensor, we determined the distance between the light source and the sensor. The linear displacement sensor can be regarded as a sliding varistor, where the movement of the sensing axis causes a change in the resistance value of the internal resistor, and the distance traveled by the sensing axis can be deduced by measuring the voltage output from the sensor. We measured the degree of the muscle relaxation of the user’s finger using a pressure sensor. In addition, during the detection process, the behavioral state of the user leads to changes in the quality of the PPG signal, such as tension changes in body posture. We used these two sensors to monitor the user’s behavioral state [21].

**Figure 2:**
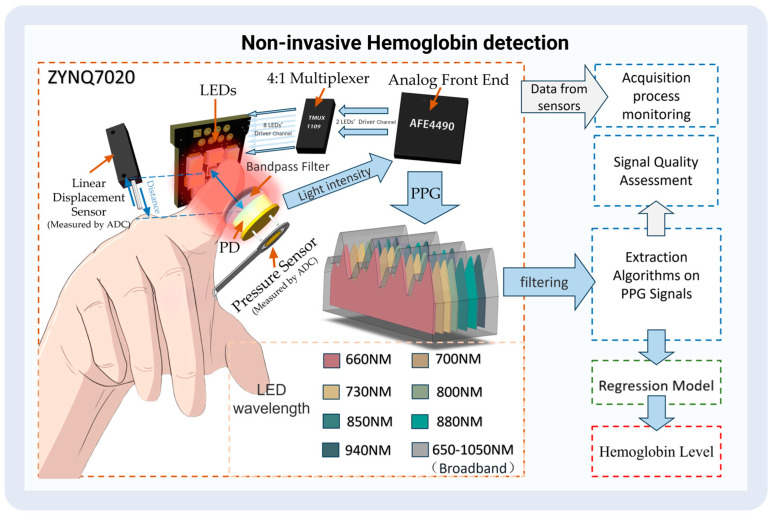
The process of non-invasive hemoglobin detection.

After collecting the data, the PPG features (the light intensity of the dynamic part and the static part) were extracted using our designed algorithm. Meanwhile, signal quality was automatically evaluated using a method we designed. Finally, the extracted PPG features were inserted into the trained regression model to determine the hemoglobin content. Figure 2 shows a workflow of the detection process.

### 3.1. Hardware Component

We used Xilinx (San Jose, CA, USA) ZYNQ7020 as the control core. According to the absorption characteristics of hemoglobin and the main interferences in the near-infrared spectral region, seven LEDs in a 2835 package from Ever-light-Electronics., Ltd. (Taiwan, China), were selected as the light source; all LEDs possessed peak wavelengths of 660 nm, 700 nm, 730 nm, 800 nm, 850 nm, 880 nm, and 940 nm bandwidths at 60 nm. We also used a broad-wavelength LED lamp bead, namely, a P1616 SFH4737 from OSRAM (Munich, Germany). The rational layout developed through multiple tests of the eight LEDs on the PCB guaranteed the highest signal-to-noise ratio. The sensor was equipped with a photodiode S1223-01 with a spectral response range of 320 nm-1100 nm from Hamamatsu (Tokyo, Japan). Also, a 550–1100 nm circular pass filter from GYTECH (Zhuji, China) was added to the photodiode to avoid interference from stray light.

The multi-wavelength pulse wave was measured using an AFE4490 dual-wavelength PPG AFE produced by TI (Dallas, TX, USA) with an acquisition frequency of 500 Hz. To enable the AFE4490 to drive eight LEDs, we added a TI (Dallas, TX, USA) TMUX1109 4:1 precision multiplexer to the AFE4490’s LED drive channel. 

**Figure 3:**
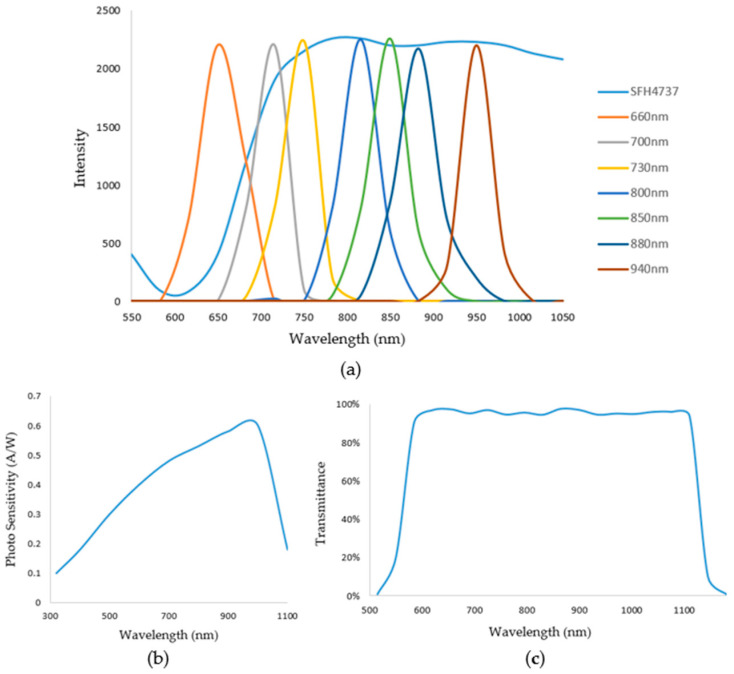
(**a**) Shows the emission spectrum. (**b**) Shows the detector spectral range sensitivity. (**c**) Shows the optical bandpass filter.

The ADC acquisition chip was 3PA1030 produced by 3PEAK (Suzhou, China), and the linear displacement sensor was a KS8 from Miran (Shenzhen, China); An FSR402 produced by Interlink Electronics (Irvine, CA, USA) was used to detect finger pressure on the sensor. The housing of the non-invasive hemoglobin instrument was also printed via 3D printing. Figure 3 illustrates a schematic of the emission spectrum, the optical bandpass filter, and the detector spectral range sensitivity.

### 3.2. Design of Finger Clip

Investigating the limitations inherent in conventional finger clips, as elucidated by Dinesh Kumar et al. [22], revealed that individual variations in finger dimensions and the presence of tremors during detection can induce alterations in a finger clip’s opening and closing angles. Such deviations subsequently impact the area of light captured by the photodiode, causing fluctuations in received light intensity. Furthermore, modifications of the range and trajectory of transmitted light through the finger contribute to variations in received transmitted light intensity and the signal quality.

**Figure 4:**
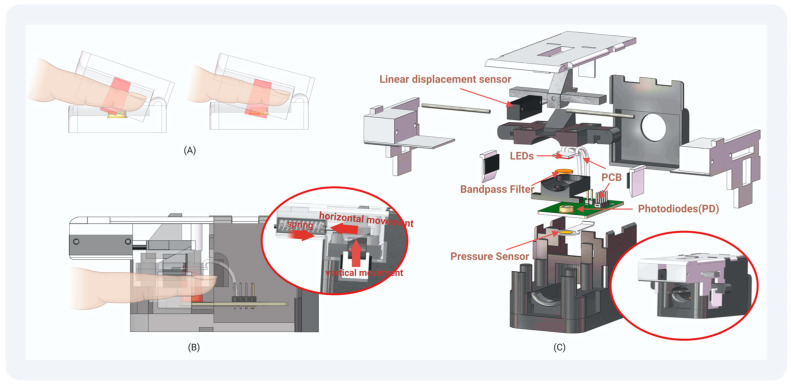
The structure of our finger clip: (**A**) Traditional Finger Clip: PD light-receiving surface changes with the opening and closing of the finger clip. (**B**) Our Finger Clip: The PD light-receiving surface does not change with the opening and closing of the finger clip. The pressure exerted by the finger and the distance from the light source to the sensor can be monitored. (**C**) Structural drawing of the finger clip we designed: Employing 3D printing techniques, we fashioned this device, allowing for assembly without the need for adhesives or screws.

Our devised finger clip design, depicted in Figure 4, integrates a PPG acquisition device, a linear displacement sensor, and a pressure sensor utilizing advanced 3D printing technology. The vertical orientation of the finger clip ensures constant inclination, thereby maintaining a consistent alignment of the light source with the photodiode. The mobile component of the finger clip allows movement of the linear displacement sensor axis mechanically, enabling the deduction of the distance between the light source and the sensor. Positioned beneath the photodiode, the pressure sensor detects the applied force from the finger. The application of Teflon tape on susceptible components minimizes wear, while light-shielding sponge material safeguards select areas from prolonged exposure for sustained stability.

**Figure 5:**
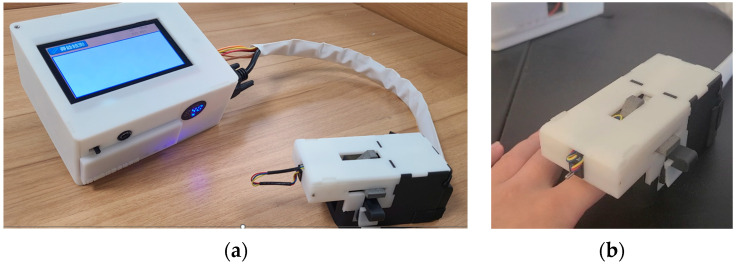
(**a**) Shows physical views of the non-invasive hemoglobin instrument. (**b**) Demonstrates a user’s application of the homemade testing finger grips.

The conceptualization of our physically non-invasive hemoglobin detector, coupled with an example of the utilization of the finger clip, is depicted in Figure 5.

## 4. PPG Signal Processing and Indicators for Signal Quality Evaluation

### 4.1. The Extraction Method for the PPG Signal Features

For the acquired PPG signals, we used a sliding average filter to filter out high-frequency noise. Then, a Chebyshev bandpass FIR filter of 0.6–10 Hz was used to filter out the baseline drift and high-frequency interference. PPG peaks and troughs were identified using morphological methods, and the difference between the peaks and neighboring troughs was calculated. Then, we arranged the values from largest to smallest according to the characteristics of the PPG, ignoring the effects of individual outliers. Because the height from the systolic blood pressure peak to the starting point is significantly greater than the height from the diastolic blood pressure peak to the notch point, the corresponding value is close to 1:1. The first half of the values was ranked from largest to smallest. Here, the portion of the larger value ranged from the height of the systolic blood pressure peak to the starting point, i.e., the dynamic portion of the PPG. For this data section, we designed a window to select the data; the window size was half of this section. We compared the variances of each window to select the window with the most minor variance and output the average value of the data in this window. We then used the sliding variance algorithm to accelerate the calculations. The recursive formulae for the sliding variance and mean are shown in Equations (6) and (7)

(6) $$\left\{ \begin{array}{l} A_{n}=\frac{1}{\Delta n}\sum_{i=n}^{n+\Delta n}X_{i}\\ A_{n}=A_{n-1}+\frac{\left(X_{n+\Delta n}-X_{n-1}\right)}{\Delta n} \end{array}\right.$$

(7) $$\left\{ \begin{array}{l} R_{n}=\frac{1}{\Delta n}{\sum_{i=n}^{n+\Delta n}\left(X_{i}-A_{n}\right)}^{2}\\ R_{n}=R_{n-1}+\frac{X_{n+\Delta n}-X_{n-1}}{\Delta n^{2}}\left\{(X_{n+\Delta n}+X_{n-1})(\Delta n-1)-2\Delta nA_{n-1}+2X_{n-1}\right\} \end{array}\right.$$

where Equation (6) is a recursive formula for the average, and ∆n represents the number of participants in the average, i.e., the window size. X represents the specific values involved in the average, and n represents the starting point of the window. $A_{n}$ represents the mean value of the nth window. Equation (7) is a recursive formula for variance, and $R_{n}$ represents the variance of the nth window.

**Figure 6:**
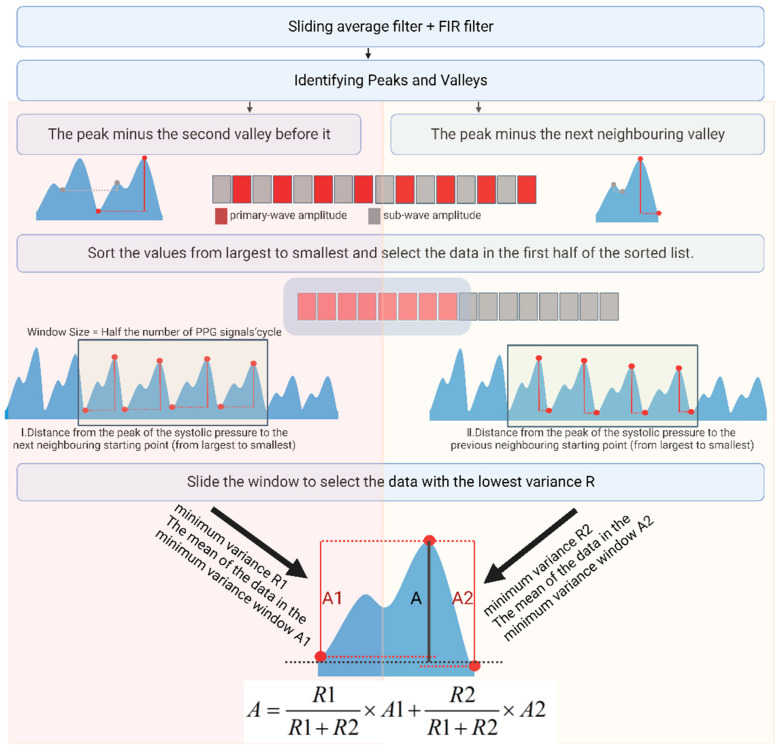
Scheme of PPG primary-wave extraction.

The height of the peak systolic pressure compared to the start of the next cycle in the PPG signal after de-baseline drift via the FIR filter may also be slightly different from the height of the peak systolic pressure compared to the beginning of the previous cycle. We extracted both amplitudes according to the sliding window variance method. We calculated the weighted average according to the variance of each amplitude to obtain the size of the dynamic portion of the PPG. The process followed for calculating the amplitude of the dynamic part of the PPG signal using the variance window is shown in Figure 6.

Following the same method, we next sorted the troughs of the PPG from largest to smallest and calculated the variance of the latter. The window with the least variance yielded the mean value of the starting point, representing the size of the static part. This method yields the most reliable data, avoids chance and error, and suppresses outliers.

### 4.2. Signal Quality Evaluation Methods

For signal quality evaluation in this study, we employed the minimum variance, indicative of the dispersion degree of the photoplethysmogram (PPG) signal amplitude, along with the count of PPG peaks and valleys, reflective of the filtering impact observed during the calculation of both dynamic and static components. These metrics were utilized as features to formulate signal quality assessment indices through the application of the AdaCost classification algorithm.

AdaCost, an algorithm rooted in AdaBoost methodology, integrates cost-sensitive principles by amalgamating multiple weak classifiers to form a robust classifier [23]. During the classification process, distinct costs were assigned to different classification errors to minimize the total cost. To establish a binary classification dataset, 100 samples from the data collection phase underwent manual annotation to distinguish signals exhibiting poor quality from those meeting the specified criteria. Cases where signals possessed poor quality but were erroneously classified as satisfactory received higher assigned costs.

The classification outcomes, as depicted in Table 1, were evaluated using three metrics, namely, recall score, precision score, and F1-score, providing insights into the efficacy of the model’s classification performance. Adaboost served as the baseline for the comparison with AdaCost in our classification approach. The AdaCost categorization yielded superior results.

## 5. Data Collection and Correlation Analysis

### Data Collection

**Figure 7:**
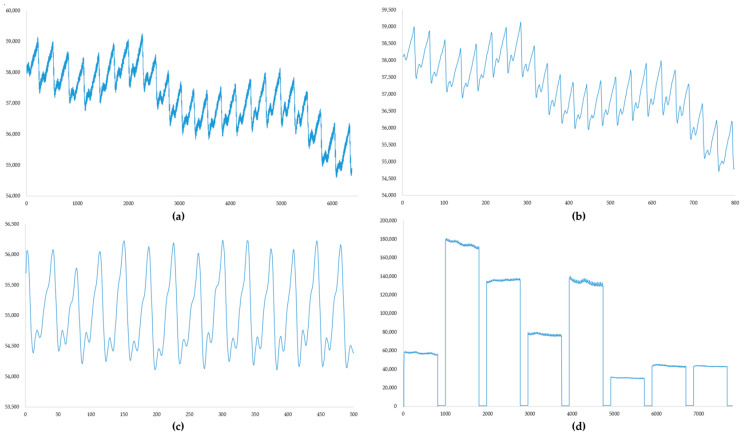
(**a**) Original PPG signal; sampling rate, 500 Hz; number of samples, 6400. (**b**) PPG signal after mean filtering. (**c**) PPG signal after filtering with an FIR filter. (**d**) The result of a single data acquisition; each PPG signal is followed by the pressure sensor data and displacement sensor data recorded in real time during the measurement.

In this study, 56 volunteers were recruited from the Department of Nephrology at the First Affiliated Hospital of Jinan University, with ages ranging from 20 to 72 years. Among the participants, 33 were male, 23 were female, and 16 were identified as anemic individuals (adult male Hb < 120 g/L; adult (non-pregnant) female Hb < 110 g/L). A non-invasive hemoglobin detector facilitated the collection of eight sets of PPG signals, complemented by data obtained from displacement and pressure sensors. Concurrently, the actual hemoglobin levels, blood pressure, creatinine, and urea levels of the volun-teers, being from the nephrology department, were recorded to explore potential in-fluences on non-invasive hemoglobin tests. During data acquisition, the volunteers reclined comfortably on a bed, placing their right index fingers into the detector’s finger clip. Measurements were conducted during stable breathing, with volunteers remaining calm throughout the process. After the test, blood collection, blood pressure testing, and the acquisition of actual hemoglobin, creatinine, urea, diastolic and systolic blood pressure, and heart rate data were conducted within a 5 min window using specialized hospital instruments. The processing flow of the pulse wave data and the results of a single acquisition are shown in Figure 7.

The filtering process illustrated in Figure 6 could be wholly implemented in the hardware used. Measurement data acquired after mean filtering were output as displayed in (d) and stored on an SD card to compile the dataset. Clinical information about the volunteers is detailed in Table 2.

## 6. Results

### 6.1. Models for Hemoglobin Level Predictions

In this investigation, we employed three prediction models: AdaBoost, a BP neural network, and Random Forest models. In the evaluation of regression prediction performance, we utilized the determination coefficient (R^2^), mean square error (MSE), and mean absolute error (MAE).

#### 6.1.1. AdaBoost Regression

AdaBoost (Adaptive Boosting) is an integrated learning method designed to combine multiple weak learners to build one strong learner [24,25]. In this paper, we used a decision tree as the base classifier. Decision trees are highly interpretable, do not necessitate the normalization of input features, are computationally undemanding, and are easy to implement quickly in hardware. The number of estimators was chosen such that the best value in the range of 1–10 and the maximum depth in the range of 1–6 could be found. We found the optimal parameters using a grid search.

#### 6.1.2. BP Neural Network

A BP neural network (BPNN) is a multi-layer feed-forward neural network that performs error correction using an error back-propagation algorithm [26]. In terms of structure, the input and output layers of the network are single-layer structures, while the hidden layers are more variable and can be single- or multi-layer structures [27]. In this study, we used a double hidden layer structure. The activation function of the network was found from among “identity”, “logistic”, “tanh”, and “real”. The activation function was optimized in terms of “identity”, “logistic”, “tanh”, and “real”, and the maximum number of iterations was 10,000, while the solver was optimized in terms of “blogs”, “sad”, and “Adam”.

#### 6.1.3. Random Forest Regression

Random Forest Regression (RF) is an integrated learning method based on decision trees. RF improves the stability and accuracy of predictions by combining predictions from multiple trees [28]. Random Forest can quantify the importance of features and guide feature selection [29]. The number of estimators was chosen such that the best value in the range of 1–10 and the maximum depth in the range of 1–6 would be found. Optimal parameters were found using a grid search.

### 6.2. Correlation Analysis

Pearson correlation analysis was employed to assess the magnitude and direction of the linear association between paired variables. This method, rooted in covariance principles, yields a correlation coefficient ranging from −1 to 1. The coefficient is computed by dividing the product of the covariance of two variables by their respective standard deviations. The proximity of the absolute value of the correlation coefficient to 1 signifies a robust linear relationship between the variables, whereas proximity to 0 indicates a weaker linear connection [30].

**Figure 8:**
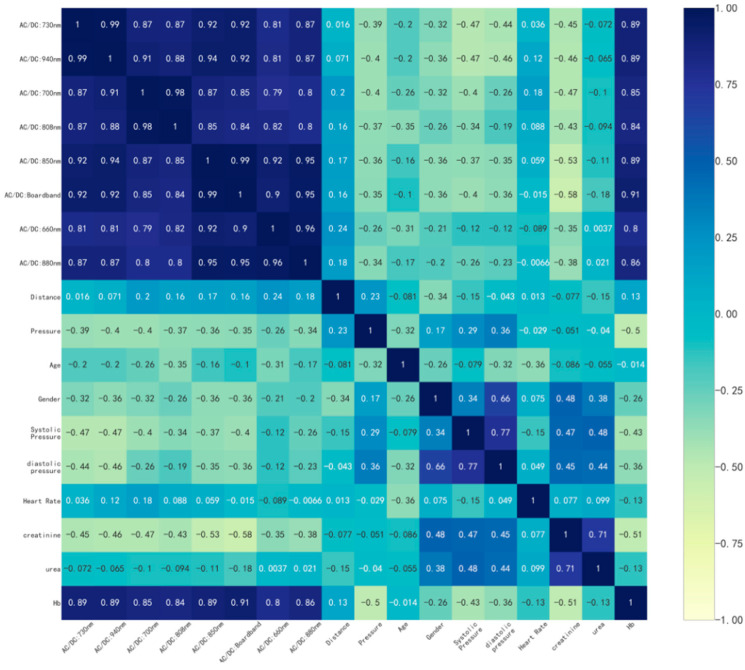
Heatmap of the correlation analysis.

Correlation analyses were performed on the collected alternative modeling parameters (creatinine, LED–sensor distance, pressure sensor data, age, gender, blood pressure, heart rate, urea, and hemoglobin). A correlation matrix of the data was calculated using Pearson’s correlation coefficient [31], and a heatmap of the correlation analysis of the data was plotted. Due to issues regarding the compliance of the volunteers, only 16 volunteers’ information (blood pressure, creatinine, and urea) was ultimately collected, and the information of these 16 volunteers was analyzed for relevant correlations. As shown in Figure 8, the ratio of the dynamic part to the static part of the PPG signal at each wavelength strongly correlated with the actual hemoglobin content. Especially for broadband LED, the correlation coefficient reached 0.91. Blood pressure, creatinine, and urea have certain correlations with hemoglobin (perhaps only for nephrotic patients), and the ratio of the dynamic part to the static part of the PPG signal measured via the broadband LED also correlates with human creatinine at an absolute value of 0.58. The LED–sensor distance has a weaker correlation with this ratio. However, the data from the pressure sensor were associated with the measurement of hemoglobin content using the PPG method.

**Figure 9:**
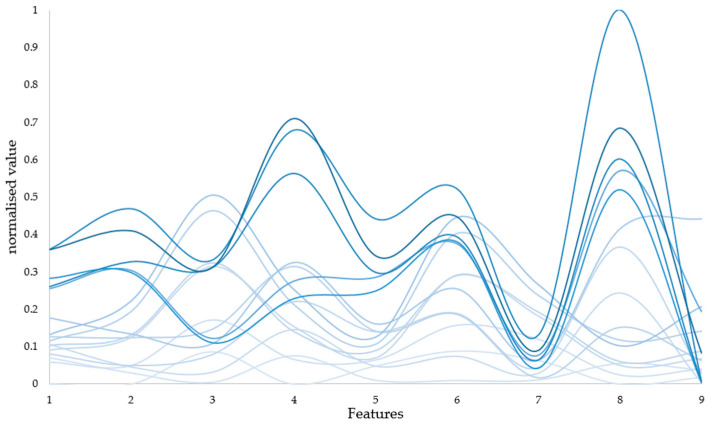
Diagram of input features. To facilitate the presentation of the obtained data, nine features were normalised. And some of the data are presented in the figure, with increased darkness of the line corresponding to elevated hemoglobin content.

Henceforth, we opted to employ the ratio of the dynamic to static components of the PPG signal across eight frequency bands, alongside the average pressure sensor data during detection, as input features, constituting a total of nine input features. Additionally, we conducted an analysis by excluding the pressure sensor data, resulting in eight input features, to discern the potential impact of pressure sensor data on prediction outcomes. Figure 9 depicts certain features within the normalized dataset, with increased darkness of the line corresponding to elevated hemoglobin content.

### 6.3. Performance Comparisons of the Models

The 56 samples were randomly divided into two groups at a ratio of 7:3, among which 17 samples were used as a test set for model validation and performance evaluation. In total, 39 samples were used as a training set to train the regression model. 

To prevent overfitting, we divided the training set using 10-fold cross-validation. We divided the training set into different subsets, excluded one subset as the validation set in each training iteration, and used the other subsets as the training set. In this way, after cycling many times, we could ensure that the samples were different each time. The average of the evaluation metrics for the test set in 10 regressions was taken as the final assessment metric. The average metrics of the three models in the 10-fold cross validation are shown in Table 3.

**Figure 10:**
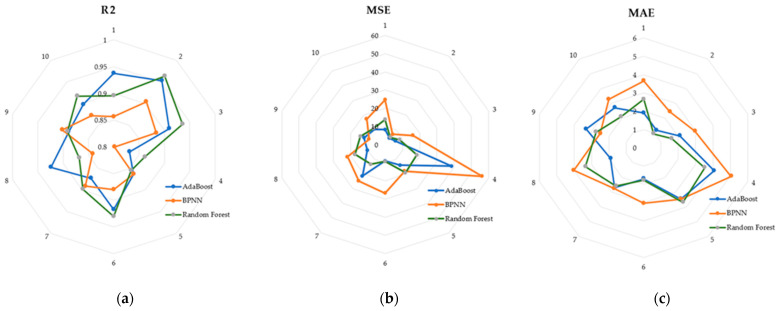
(**a**) R^2^ of the three models in the cross-validation; (**b**) MSE of the three models in the cross-validation; (**c**) MAE of the three models in the cross-validation.

Random forests achieved better performance in the test set, and we used the radar charts shown in Figure 10 to show in detail the performance of the three models in the 10-fold cross-validation. Each vertex of the radar plot corresponds to 1 fold in the 10-fold cross-validation. Each model achieves an R^2^ of 0.8 or more in each fold.

The application of K-fold cross-validation is restricted to validation purposes and does not serve as a foundation for conclusive model parameter judgments. Nevertheless, it proves invaluable for discerning optimal hyperparameter combinations, facilitating structural adjustments to a model, and subsequently reinitializing the model for enhanced parameterization. Ultimately, the model undergoes a comprehensive re-training on the entire dataset, utilizing the optimal hyperparameters, and the resultant generalization performance is evaluated through assessment scores on the test dataset.

The model’s training parameters encompassed the dynamic-to-static ratio of the PPG signal across eight bands and the mean value of pressure sensor data during detection. Additionally, we conducted a comparative analysis by excluding pressure sensor data, thereby forming a dataset consisting of eight features. Table 4 presents the statistical analysis values for the proposed models.

As shown in Table 4, all three models showed better results with the addition of data from the pressure sensor. Among them, the AdaBoost model was the most effective, with an R^2^ of 0.91, an MSE of 13.99, and an MAE of 2.67.

**Figure 11:**
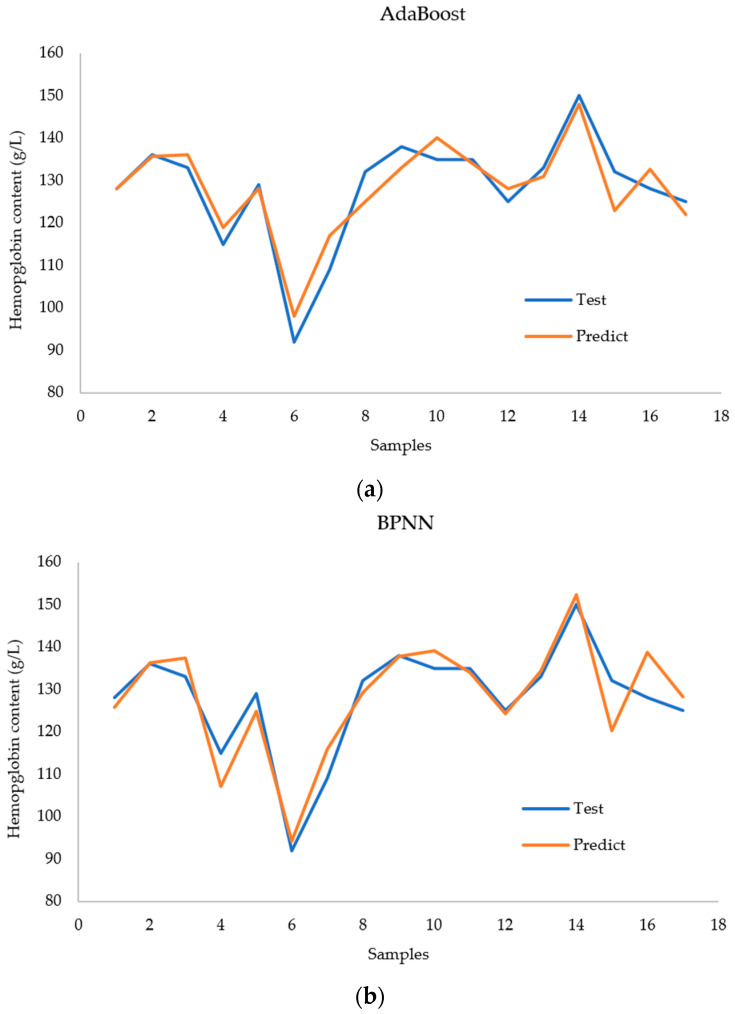

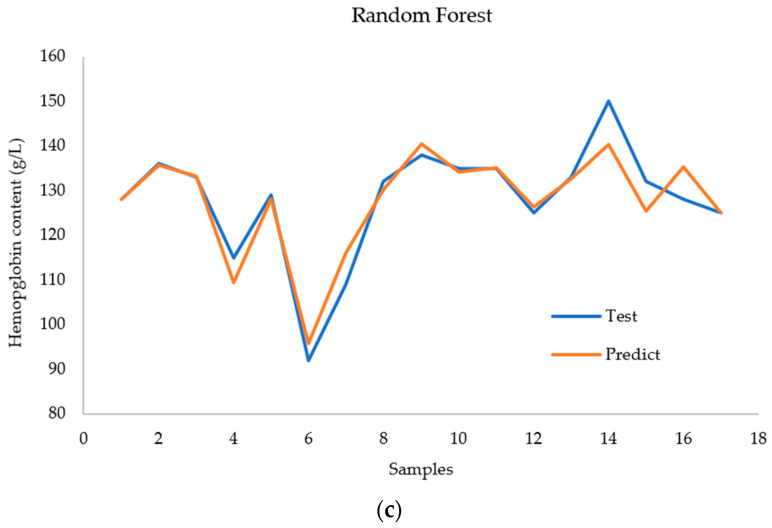
Comparison of (**a**) the AdaBoost regression model, (**b**) the BPNN regression model, and (**c**) the RF regression model with regard to predicting hemoglobin concentration.

Figure 11 shows a comparison between the actual values and the prediction results of each model for the case with nine inputs. The AdaBoost model achieved better performance for all the test samples. The BPNN model had large deviations in the 4th, 6th, and 17th test samples. The random forest model performed well for all samples except the 14th test sample. However, the predicted values deviate significantly from the true values of the 14th sample.

**Figure 12:**
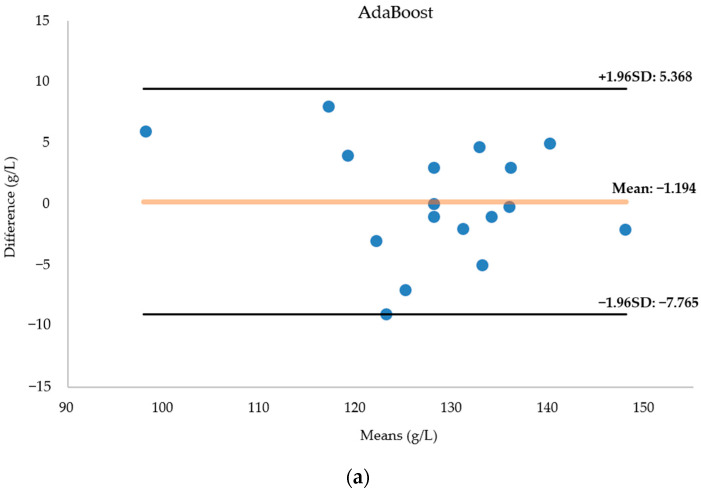

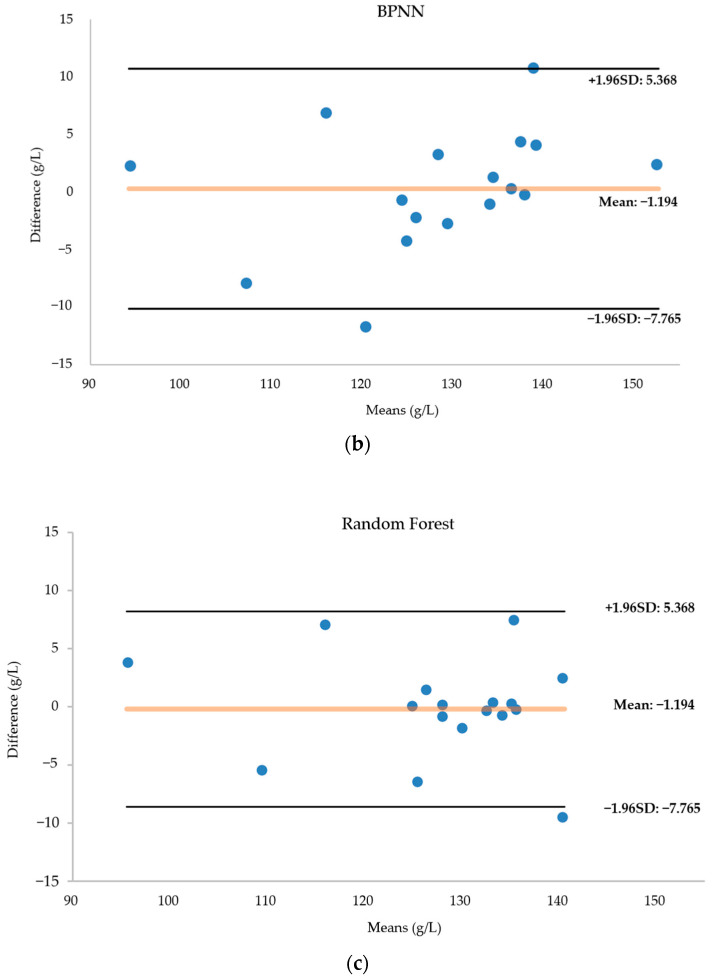
(**a**) Bland–Altman diagram of the AdaBoost regression model for predicting hemoglobin concentration. (**b**) Bland–Altman diagram of the BPNN regression model for predicting hemoglobin concentration. (**c**) Bland–Altman diagram of the RF regression model for predicting hemoglobin concentration. The horizontal coordinate of the Bland-Altman diagram is the mean of the predicted and actual values, the vertical coordinate is the difference between the predicted and actual values, and the diagram in-cludes three lines, with the middle line indicating the mean of the difference, and the top and bottom lines being the upper and lower limits of the 95 per cent consistency boundaries. The Bland-Altman diagram allows visualisation of the measurement deviation and degree of consistency between two observations. If most of the points are distributed near the mean difference and within the standard deviation, the two data sets are in good agreement.

Figure 12 presents a Bland–Altman diagram for the three regression models fore-casting hemoglobin concentration. Notably, the AdaBoost model’s predictions closely align with the actual values, with no data surpassing the 95 percent consistency threshold. The AdaBoost prediction model emerges as the most suitable for modeling this dataset, showcasing superior predictive capabilities. In contrast, the BPNN results exhibit more deviations from the actual values, with two data points falling outside acceptable bounds. While the random forest model accurately predicts most data points, seven predictions significantly deviate from reality.

## 7. Discussion

The Lambert–Beer Law served as the foundational theoretical framework for our non-invasive hemoglobin detection approach, employing eight bands of LEDs as an illumination source. A specially designed finger clip, along with additional features, was implemented to mitigate potential interference factors during testing. Our experimental observations revealed that the spacing between the light source and the sensor had a minimal impact on predictions. This effect appeared to be mitigated by the division of the dynamic and static components of the measured pulse wave, serving as a basis for signal quality assessment. Finger pressure emerged as a notable influencer, evident in both correlation analyses and prediction outcomes. The pliability of human fingers and the resulting diverse deformations may affect parameters such as muscle contraction and the vascular perfusion index, warranting further experimentation for comprehensive validation. Notably, our exploration unveiled a superior noninvasive detection technique utilizing broadband light sources, with potential applications in creatinine detection. Our study harnessed three regression models, all demonstrating commendable predictive outcomes and validating our conceptual framework. However, further data accumulation, diverse population experimentation, and the identification of additional influencing factors are imperative for refining the efficacy of our device.

## 8. Conclusions

In this study, we designed a device for measuring hemoglobin based on the properties of PPG. The measurement of hemoglobin is based on PPG’s ability to respond to the absorption of arterial blood components. We used eight bands of LEDs as light sources for PPG acquisition, including a broadband LED. A finger clip was designed to acquire more features and achieve better immunity to interference. The most appropriate PPG period was selected using a variance window, and features were computed while the quality of the signal was classified via the AdaCost algorithm. In addition, we recruited 38 volunteers for clinical trials to obtain accurate data. In the correlation study, we found that blood pressure and creatinine levels may interfere with the prediction of hemoglobin levels using NIR PPG features in patients with kidney disease. Adding a pressure sensor to the bottom of the PD and using a broadband LED facilitated the prediction of hemoglobin content. The results showed that the AdaBoost model offered the best effects, with an R^2^ of 0.91, an MSE of 13.99, and an MAE of 2.67. The proposed device and method have great potential for monitoring hemoglobin concentrations.

In the future, we need to collect more data to train the model to achieve better results. We also need to find features or measurements with greater correlation for non-invasive hemoglobin testing, improving the vital recognition ability of our detection system. In addition, based on the results of the correlation analysis, we will further investigate the non-invasive measurement of other critical physiological indicators such as creatinine and blood pressure. Wearable devices are also an important direction of development and could include flexible electronics [32], triboelectric nanogenerators [33], inkjet printing, and other technologies, which will make non-invasive body-parameter-testing devices more conducive to long-term monitoring.

## Figures and Tables

**Table 1:** Classification results of AdaBoost and AdaCost.

Classification Model	Recall Score *	Precision Score *	F1 Score *
AdaBoost	0.83	0.75	0.79
AdaCost	0.94	0.89	0.92

* Recall score: Recall quantifies the number of positive class predictions made out of all positive examples in the dataset. Precision score: Precision quantifies the number of positive class predictions that actually belong to the positive class. F1 score: The harmonic mean of precision and recall.

**Table 2:** Summary tables including all the relevant clinical characteristic.

NO.	Hemoglobin Content (g/L)	Gender	Age	Blood Pressure (mmHg) Diastolic/Systolic	Heart Rate (BPM)	Creatinine (μmoI/L)	Urea (mmol/L)
1	135	Male	23				
2	128	Female	25				
3	130	Male	72	129/80	70	94.8	8.09
4	136	Male	21				
5	110	Male	48	147/97	79	67.3	2.91
6	148.5	Male	26				
7	147.9	Male	36				
8	119	Female	30	165/107	70	173.8	8.92
9	136.5	Male	25				
10	117	Male	36	129/102	93	229.8	10.34
11	133	Male	24				
12	118	Male	38	160/98	75	360.2	27.17
13	92	Male	38				
14	125	Female	28				
15	133	Female	64	120/76	85	66.3	5.38
16	124	Female	55	155/84	63	61.1	5.16
17	106	Male	58	141/93	63	551.3	29.5
18	119	Male	24				
19	131	Male	26				
20	124	Female	25	120/80	86	90	4
21	150	Male	28				
22	88	Male	27	152/92	84	207.1	13.07
23	102.5	Male	48				
24	126	Female	26				
25	133	Female	24				
26	115	Male	40				
27	140	Female	41				
28	129.7	Female	30				
29	132.7	Male	24				
30	103	Female	33				
31	109	Male	30				
32	132	Female	24				
33	122	Male	36				
34	123	Female	39				
35	134.6	Male	25				
36	132	Male	26				
37	118	Male	35				
38	138	Female					
39	127	Male	26				
40	132	Female	23				
41	128	Female	39	117/63	70	67.3	2.91
42	129	Female	25				
43	128	Female	27				
44	138	Male	24	110/80	70	90	4
45	136	Male	27	155/110	60	178.4	9.04
46	133	Male	20	160/101	97	344.1	34.16
47	136	Male	36				
48	135	Male	24	110/80	88	90	4
49	125	Female	28				
50	125	Female					
51	134	Male	44				
52	127.9	Female	55	125/74	68		
53	127	Female	36				
54	131	Male	24				
55	129	Female	25				
56	96	Male	32	152/92	86	474.6	4.29

**Table 3:** Performance of three models in cross-validation.

Classification Model	R^2^	MSE	MAE
AdaBoost	0.89	13.43	2.50
BP neural network	0.86	22.21	3.32
Random Forest	0.90	13.02	2.48

**Table 4:** The performance of the three models in statistical analysis.

Classification Model	R^2^		MSE		MAE	
	Nine Inputs	Eight Inputs	Nine Inputs	Eight Inputs	Nine Inputs	Eight Inputs
AdaBoost	0.91	0.86	13.99	20.78	2.67	3.35
BP neural network	0.87	0.82	24.64	26.86	3.77	3.91
Random Forest	0.89	0.85	15.20	22.92	2.80	3.15

## Data Availability

Data are contained within the article.

## References

[1] Bell S.G. (1999). An introduction to hemoglobin physiology. Neonatal Netw..

[2] Topal M., Guney I. (2023). The association of soluble Klotho levels with anemia and hemoglobin variability in hemodialysis patients. Semin. Dial..

[3] Mutonhodza B., Dembedza M.P., Lark M.R., Joy E.J.M., Manzeke-Kangara M.G., Njovo H., Nyadzayo T.K., Kalimbira A.A., Bailey E.H., Broadley M.R. (2023). Anemia in children aged 6-59 months was significantly associated with maternal anemia status in rural Zimbabwe. Food Sci. Nutr..

[4] Noshiro K., Umazume T., Hattori R., Kataoka S., Yamada T., Watari H. (2022). Hemoglobin Concentration during Early Pregnancy as an Accurate Predictor of Anemia during Late Pregnancy. Nutrients.

[5] Mannucci Tragodara S.M., Del Aguila Villar C.M., Rojas Gabulli M.I., Falen Boggio J.M., Lu de lama L.R., Nunez Almache O., Chavez Tejada E.M.D.J., Espinoza Robles O.A., Pinto Ibarcena P.M., Calagua Quispe M.R. (2021). Anemia due to Iron Deficiency and Relationship with Glycosylated Hemoglobin Levels in Diabetic Children. Horm. Res. Paediatr..

[6] Napolitano L.M. (2021). Vitamin D supplementation and hemoglobin: Dosing matters in prevention/treatment of anemia. Nutr. J..

[7] Joseph B., Haider A., Rhee P. (2016). Non-invasive hemoglobin monitoring. Int. J. Surg..

[8] Allen J. (2007). Photoplethysmography and its application in clinical physiological measurement. Physiol. Meas..

[9] Liu A., Li G., Yan W., Lin L. (2018). Combined effects of PPG preprocess and dynamic spectrum extraction on predictive performance of non-invasive detection of blood components based on dynamic spectrum. Infrared Phys. Technol..

[10] Conley C.L. (1964). Pathophysiological Effects of Some Abnormal Hemoglobins. Medicine.

[11] Kumar Y., Dogra A., Kaushik A., Kumar S. (2022). Progressive evaluation in spectroscopic sensors for non-invasive blood haemoglobin analysis—A review. Physiol. Meas..

[12] Yuan J., Ding H., Gao H., Lu Q. (2015). Research on improving the accuracy of near infrared non-invasive hemoglobin detection. Infrared Phys. Technol..

[13] Ahsan G.M.T., Gani M.O., Hasan M.K., Ahamed S.I., Chu W., Adibuzzaman M., Field J. A Novel Real-Time Non-Invasive Hemoglobin Level Detection Using Video Images from Smartphone Camera. Proceedings of the 41st IEEE Annual Computer Software and Applications Conference (COMPSAC).

[14] Kesarwani A., Das S., Dalui M., Kisku D.R., Sen B., Roy S., Basu A. (2023). Non-invasive anaemia detection by examining palm pallor: A smartphone-based approach. Biomed. Signal Process. Control.

[15] Kumar Y., Dogra A., Shaw V., Kaushik A., Kumar S. (2022). NIR-based Sensing System for Non-invasive Detection of Hemoglobin for Point-of-care Applications. Curr. Med. Imaging.

[16] Kavsaoglu A.R., Polat K., Hariharan M. (2015). Non-invasive prediction of hemoglobin level using machine learning techniques with the PPG signal’s characteristics features. Appl. Soft Comput..

[17] Kraitl J., Timm U., Ewald H. Non-invasive measurement of blood and tissue parameters based on VIS-NIR spectroscopy. Proceedings of the Conference on Optical Diagnostics and Sensing XIII—Toward Point-of-Care Diagnostics.

[18] Wieringa F.P., Mastik F., van der Steen A.F.W. (2005). Contactless multiple wavelength photoplethysmographic imaging: A first step toward “SpO(2) camera” technology. Ann. Biomed. Eng..

[19] Landsman M.L., Kwant G., Mook G.A., Zijlstra W.G. (1976). Light-absorbing properties, stability, and spectral stabilization of indocyanine green. J. Appl. Physiol..

[20] Reiser M., Breidenassel A., Amft O., IEEE Simulation framework for reflective PPG signal analysis depending on sensor placement and wavelength. Proceedings of the 4th IEEE-EMBS International Conference on Wearable and Implantable Body Sensor Networks (BSN)/18th IEEE-EMBS International Conference on Biomedical and Health Informatics (BHI).

[21] Teng X.F., Zhang Y.T. (2004). The effect of contacting force on photoplethysmographic signals. Physiol. Meas..

[22] Kumar R.D., Guruprasad S., Kansara K., Rao K.N.R., Mohan M., Reddy M.R., Prabhu U.H., Prakash P., Chakraborty S., Das S. (2021). A Novel Noninvasive Hemoglobin Sensing Device for Anemia Screening. IEEE Sens. J..

[23] Fan W., Stolfo S.J., Zhang J.X., Chan P.K. AdaCost: Misclassification cost-sensitive boosting. Proceedings of the 16th International Conference on Machine Learning (ICML 99).

[24] Zhu J., Zou H., Rosset S., Hastie T. (2009). Multi-class AdaBoost. Stat. Its Interface.

[25] Ramakrishna M.T., Venkatesan V.K., Izonin I., Havryliuk M., Bhat C.R. (2023). Homogeneous Adaboost Ensemble Machine Learning Algorithms with Reduced Entropy on Balanced Data. Entropy.

[26] Wang H., Li G., Zhao Z., Lin L. Dynamic Spectrum and BP Neural Network for Non-invasive Hemoglobin Measurement. Proceedings of the International Conference on Life System Modeling and Simulation/International Conference on Intelligent Computing for Sustainable Energy and Environment (LSMS-ICSEE).

[27] Ben S.J., Dorner M., Gunther M.P., von Kanel R., Euler S. (2023). Proof of concept: Predicting distress in cancer patients using back propagation neural network (BPNN). Heliyon.

[28] Breiman L. (2001). Random forests. Mach. Learn..

[29] Austin A.M., Ramkumar N., Gladders B., Barnes J.A., Eid M.A., Moore K.O., Feinberg M.W., Creager M.A., Bonaca M., Goodney P.P. (2022). Using a cohort study of diabetes and peripheral artery disease to compare logistic regression and machine learning via random forest modeling. Bmc Med. Res. Methodol..

[30] Rovetta A. (2020). Raiders of the Lost Correlation: A Guide on Using Pearson and Spearman Coefficients to Detect Hidden Correlations in Medical Sciences. Cureus.

[31] Chaila M.Z., Viniegra M., Gagliardino J.J., Martinez A., Simesen de Bielke M.G., Frusti M., Monaco L., Salgado P., Buso C., Gonzalez C.D. (2022). Glycated Hemoglobin Measurement: Comparison of Three Methods Versus High Performance Liquid Chromatography. J. Diabetes Sci. Technol..

[32] Zhu W.W., Yu H.X., Pu Z.H., Guo Z.J., Zheng H., Li C.C., Zhang X.G., Li J., Li D.C. (2023). Effect of interstitial fluid pH on transdermal glucose extraction by reverse iontophoresis. Biosens. Bioelectron..

[33] Zhang C., Zhang L., Pu Z.H., Bao B., Ouyang W.Y., Li D.C. (2023). Fabricating 1D stretchable fiber-shaped electronics based on inkjet printing technology for wearable applications. Nano Energy.

